# The Evolution of Insulin and How it Informs Therapy and Treatment Choices

**DOI:** 10.1210/endrev/bnaa015

**Published:** 2020-05-12

**Authors:** Irl B Hirsch, Rattan Juneja, John M Beals, Caryl J Antalis, Eugene E Wright

**Affiliations:** 1 University of Washington Diabetes Institute, Seattle, Washington; 2 Eli Lilly and Company, Lilly Corporate Center, Indianapolis, Indiana; 3 Eli Lilly and Company, Lilly Biotechnology Center, San Diego, California; 4 Charlotte Area Health Education Center, Charlotte, North Carolina

**Keywords:** insulin, rapid-acting, long-acting, pharmacokinetics, pharmacodynamics

## Abstract

Insulin has been available for the treatment of diabetes for almost a century, and the variety of insulin choices today represents many years of discovery and innovation. Insulin has gone from poorly defined extracts of animal pancreata to pure and precisely controlled formulations that can be prescribed and administered with high accuracy and predictability of action. Modifications of the insulin formulation and of the insulin molecule itself have made it possible to approximate the natural endogenous insulin response. Insulin and insulin formulations had to be designed to produce either a constant low basal level of insulin or the spikes of insulin released in response to meals. We discuss how the biochemical properties of endogenous insulin were exploited to either shorten or extend the time-action profiles of injectable insulins by varying the pharmacokinetics (time for appearance of insulin in the blood after injection) and pharmacodynamics (time-dependent changes in blood sugar after injection). This has resulted in rapid-acting, short-acting, intermediate-acting, and long-acting insulins, as well as mixtures and concentrated formulations. An understanding of how various insulins and formulations were designed to solve the challenges of insulin replacement will assist clinicians in meeting the needs of their individual patients.

## Graphical Abstract

**Graphical Abstract:**
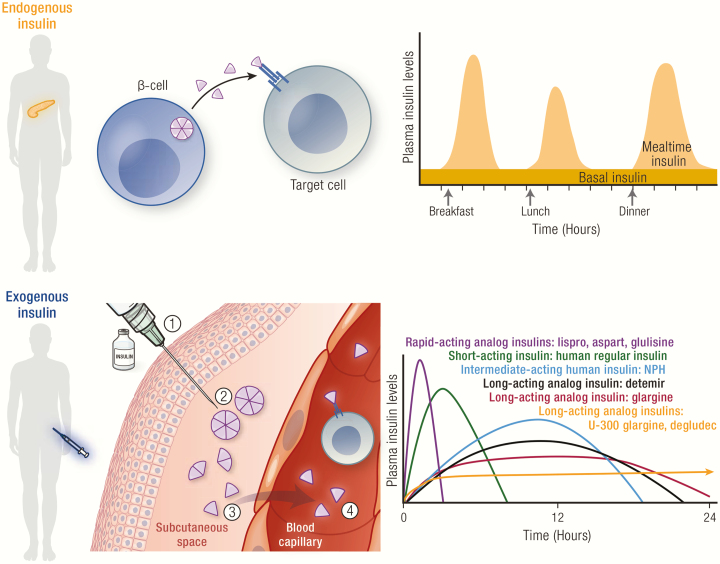

**Essential points**

- Insulin has been available for the treatment of diabetes for almost 100 years.
- Therapeutic insulin has evolved from a crude extract of animal pancreas to recombinant human insulin and insulin analogs.
- The time-action profiles of insulins and formulations have been intentionally modified to more closely mimic the endogenous insulin response.
- Endogenous insulin in the pancreas forms hexamers–6 insulin molecules held together by intermolecular interactions and zinc ions–which dissolve into active monomers in the blood stream.
- Varying the insulin formulation and the insulin molecule to affect hexamer formation is a key to speeding or slowing the absorption of injected insulin into the circulation.
- The pharmacokinetic and pharmacodynamic profiles of various insulins illustrate the effects of these modifications.

## History of Insulin Development

The 100^th^ anniversary of the discovery and commercial availability of insulin as a treatment for diabetes is approaching. The discovery of insulin is attributed to a group at the University of Toronto led by J. J. R. Macleod, a professor of physiology. In 1921, Macleod accepted a proposal by Frederick G. Banting, a 22-year-old physician and surgeon, to work in his laboratory to test his ideas on pancreatic extracts for reducing blood glucose in diabetic dogs. Banting, assisted by summer student Charles Best, began to accumulate evidence that these extracts worked. J.B. Collip, a visiting biochemist, joined the group and provided the expertise needed to purify the active glucose-lowering component from the extracts, leading to the first successful test of insulin in a 14-year-old boy with diabetes, Leonard Thompson, in January 1922. Key developments from other labs and the pharmaceutical industry allowed for the large-scale commercial production of insulin by the end of 1923 (1).

**Figure 1.**
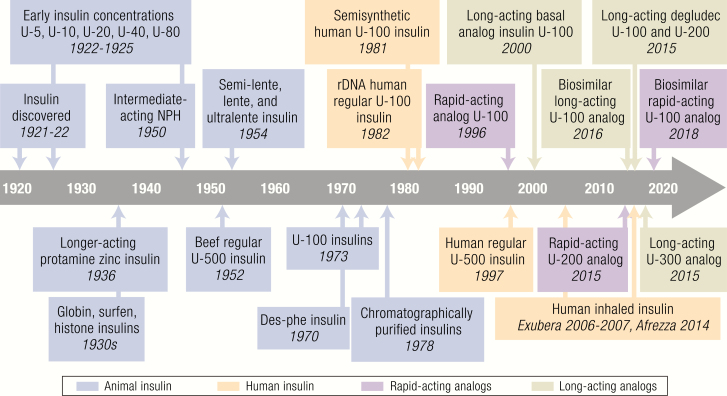
Timeline of insulin development with approximate historical dates. Abbreviations: NPH, neutral protamine Hagedorn; rDNA, recombinant DNA; U = units.

This last century has been a time of change and innovation in the field of insulin therapy, starting with the isolation of insulin, the purification and concentration of animal pancreatic extracts, the development of formulations with protracted duration of action, and the progression to human insulin and modified insulin analogs made with recombinant DNA technology. The landscape of insulins available today also includes insulin mixtures, concentrated insulins, and insulins with alternate routes of administration, providing a wide array of options for people living with diabetes (Fig. 1).

Exogenous insulins are now available as rapid-acting, short-acting, intermediate-acting, and long-acting. The timing of insulin action arises from the unique pharmacokinetics (PK) and pharmacodynamics (PD) of each insulin and/or insulin formulation. Pharmacokinetics refers to the time course of the circulating concentration of insulin that results from a subcutaneous (SC) injection or other method of delivery. Pharmacodynamics refers to the time course of the effect on blood glucose concentration. Because all insulins have wide-ranging effects on the body, they must be prescribed with an understanding of the PK and PD to have an optimal effect (ie, maintaining blood glucose in the normal range) without endangering the patient (ie, hypoglycemia).

Both PK and PD are affected by the characteristics of the insulin injected and a range of physiological factors, including exercise, body temperature, and insulin sensitivity. An understanding of the PK and PD characteristics of the various insulins and/or insulin formulations, as well as physiological factors that can modify PK and PD, is an essential starting point in being able to confidently prescribe an insulin regimen.

This review discusses how insulin works, both naturally and when injected, and how its mode of action was exploited in the design of synthetic molecules with varying PK profiles that can therapeutically mimic the body’s natural insulin responses. The PK and PD profiles of these synthetically created insulins are shown herein to illustrate their unique actions and to facilitate the tailoring of insulin therapy to meet the needs of the individual patient.

## Insulin Structure and Chemistry

**Figure 2.**
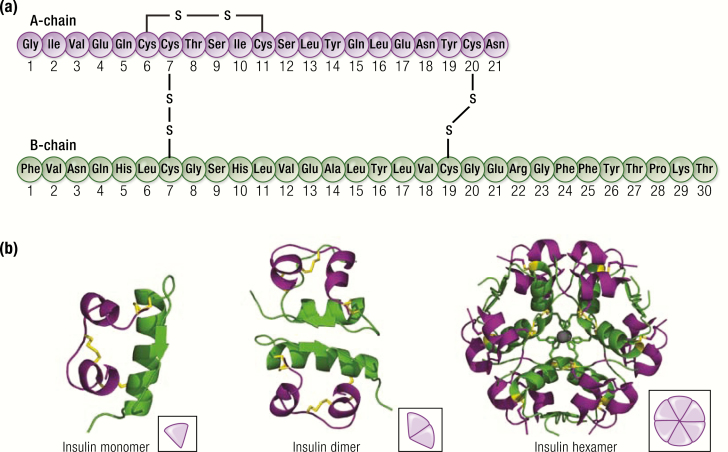
Structure of human insulin. **A:** Amino acid sequence of human insulin. **B:** Three-dimensional structure of insulin monomer (A-chain in purple; B-chain in green; protein data bank [PDB] ID = 1LPH), insulin dimer (PDB ID = 1LPH), and insulin hexamer (comprised of 3 dimers and 2 Zn2+) (PDB ID = 2INS). (https://www.rcsb.org/pdb/static.do?p=general_information/about_pdb/index.html); 1LPH, 2INS=PDB ID for respective structures)

The basis of all modern insulin analogs/derivatives and insulin formulations is human insulin, a small protein of 51 amino acids consisting of 2 chains, the A-chain, composed of 21 amino acids, and the B-chain, composed of 30 amino acids. Two interchain disulfide bridges (Cys^A7^ to Cys^B7^ and Cys^A20^ to Cys^B19^) covalently link chains A and B. Chain A also contains an intrachain (Cys^A6^ to Cys^A11^) disulfide bridge (Fig. 2A) (2). In the pancreatic β-cells, the active 2-chain insulin molecule is generated from a single-chain proinsulin precursor by the proteolytic excision and release of C-peptide, which circulates and is used as a measure of endogenous insulin secretion. Proinsulin is secreted at the same time as insulin, but has low biological activity (3).

**Figure 3.**
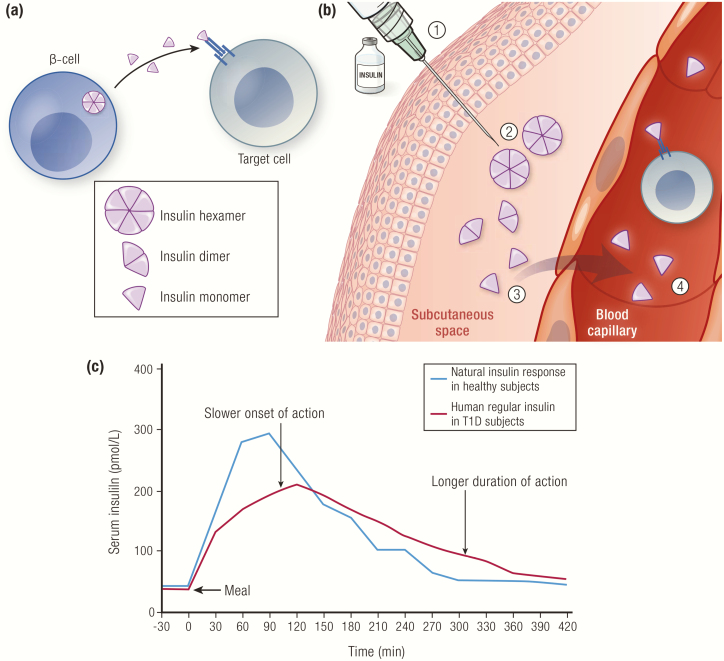
Role of insulin hexamers in insulin PK. **A:** Insulin hexamers in pancreatic β-cells disassemble into monomers upon dilution in the blood stream and diffuse into tissues where they bind to insulin receptors on target cells. **B:** (1) Regular insulin is formulated as hexamers in the presence of zinc; (2) insulin is injected into the SC space; (3) insulin hexamers dissociate into dimers then monomers in the SC space; (4) the size of the monomers permits absorption across the microvascular endothelium into the blood. **C:** Pharmacokinetics profile of endogenous insulin (blue line) following a meal in healthy subjects (n = 6), and of exogenous insulin (red line) when human regular insulin (0.1 U/kg SC) was injected 5 minutes before the meal in subjects with type 1 diabetes (n = 6). Data from Pampanelli S, Torlone E, Ialli C, Del Sindaco P, Ciofetta M, Lepore M, et al. Improved postprandial metabolic control after subcutaneous injection of a short-acting insulin analog in IDDM of short duration with residual pancreatic beta-cell function. *Diabetes Care.* 1995;18(11):1452–1459.

Insulin is stored within the pancreatic β-cells as hexamers stabilized by zinc ions; 6 monomers readily form 3 dimers that assemble into hexamers in the presence of zinc (Fig. 2B) (4). When secreted from the β-cells, the zinc-insulin hexamers are diluted in the blood stream, causing the zinc to be released, which results in the hexamers disassembling into monomers—the active state of insulin (Fig. 3A). The blood-borne monomers initially perfuse the liver and kidney, where a significant portion are cleared, and active insulin works to suppress liver glycogenolysis and gluconeogenesis. Ultimately, the insulin monomers are carried to the peripheral tissues where they act primarily on muscle and fat, imparting a variety of biochemical processes, including stimulation of glucose uptake, inhibition of adipose triglyceride breakdown, and fuel utilization.

In exogenous insulin formulations, hexamer formation can be triggered by the addition of special additives to form stable solutions for vials and cartridges. Classic additives are zinc, a phenolic preservative (m-cresol and/or phenol) that serves a dual purpose as an antibacterial agent and a hexameric stabilizer, and a buffer to maintain the correct pH. When the insulin in these formulations is injected into the SC space, dilution of the additives in the interstitial fluid will cause the hexamers to disperse into monomers and enter the blood stream (Fig. 3B).

The first commercial insulin formulations were made with animal insulins, primarily beef and pork insulins, which had PK and PD properties very similar to those of human insulin in spite of differences in their amino acid sequences (5). A common problem with animal-source insulins, however, was the formation of anti-insulin antibodies, which led to lipoatrophy and insulin resistance in a significant percentage of patients (6, 7). To address this problem, chromatographic processing techniques were developed to purify active insulin from proinsulin and other immunogenic peptides, resulting in “monocomponent” or “single peak” insulin in the 1970s (8). Porcine des-phe insulin, where the N-terminal phenylalanine was removed from the B-chain (9), and a semisynthetic human insulin, made by the enzymatic substitution of 1 amino acid in pork insulin (10–12), were developed in the 1980s. These insulins were less immunogenic but biological activity was only marginally better than their unmodified counterparts (7, 13).

As demand for insulin grew, and with a limited supply of animal pancreata in some countries, there was a need for a scalable insulin source. The discovery of the insulin gene and commercialization of recombinant DNA technology enabled the development and large-scale manufacturing of biosynthetic human insulin (14, 15). The first biosynthetic human insulin product was approved in 1982, marketed under the brand name Humulin^®^ R (Eli Lilly and Company, Indianapolis, IN) (16). It was followed by Novolin^®^ R (Novo Nordisk A/S, Bagsvӕrd, Denmark) in 1991 (17) and Insuman^®^ R (Hoechst, Frankfurt, Germany) in 1997. The advent of human insulin led to the decline in the use of the animal-based products, which were subsequently removed from the market in most countries. Even with human insulin, low titers of anti-insulin antibodies still appear in most patients but are without consequence.

## Time-action Profile of Insulin

**Figure 4.**
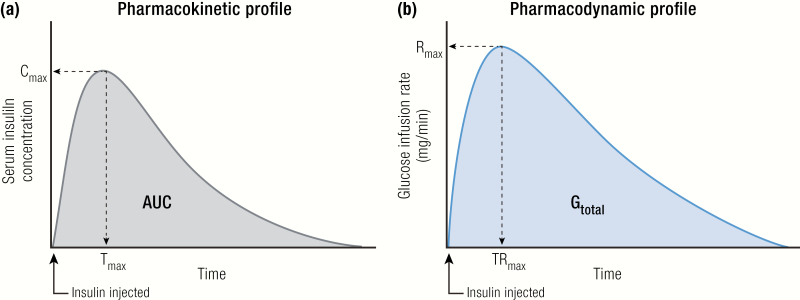
Pharmacokinetics and PD profiles, explained. **A:** Pharmacokinetics profile: insulin concentration versus time after injection. **B:** Pharmacodynamics profile: GIR versus time after insulin injection. Abbreviations: AUC, area under the curve; C_max_, maximum concentration reached; G_total_, total amount of glucose infused; R_max_, maximum rate of glucose infusion; T_max_, time to reach C_max_; TR_max_, time to reach R_max_.

The time-action profile of insulin is measured in carefully controlled clinical pharmacology studies. In these studies, fasted participants are given an insulin dose in coordination with a meal. The curve of the measured insulin concentration in the blood versus time is the PK profile (Fig. 4A), and the curve of the blood glucose concentration versus time is the PD profile. In a euglycemic clamp study, instead of a meal, glucose is infused intravenously at a variable rate to keep blood glucose levels constant after the insulin dose is given. The glucose infused per unit of time is called the glucose infusion rate (GIR), and the curve of the GIR versus time is the PD profile (Fig. 4B). Under the controlled conditions of the euglycemic clamp, the PD profile of the insulin being tested will mimic the PK profile (18, 19).

In real life, physiological factors can create a significant between-day and between-individual variation in the time to onset and peak action. These factors, which are not mutually exclusive, include body temperature, physical activity, blood flow at the injection site, the location of the injection site, the presence of lipodystrophy at the injection site, and insulin sensitivity. But under the controlled conditions of a trial, and with enough subjects, a good representation of the PK and PD profiles of an insulin can be established with these methods.

The pharmacometric profiles are used to define 3 features of the time-action profile: (1) onset of action, defined as the time after injection when blood glucose-lowering activity is observed; (2) time to peak, defined as time after the injection to reach maximum effect; and (3) duration of action, defined as how long after injection the insulin action lasts.

### What causes the time-action profile to vary?

By design, the time-action profiles of exogenous insulins and/or insulin formulations differ from each other; the onset, peak, and duration of action have been modulated to better mimic natural insulin release profiles. This works through a time-dependent insulin association/dissociation process that occurs after injection.

**Figure 5.**
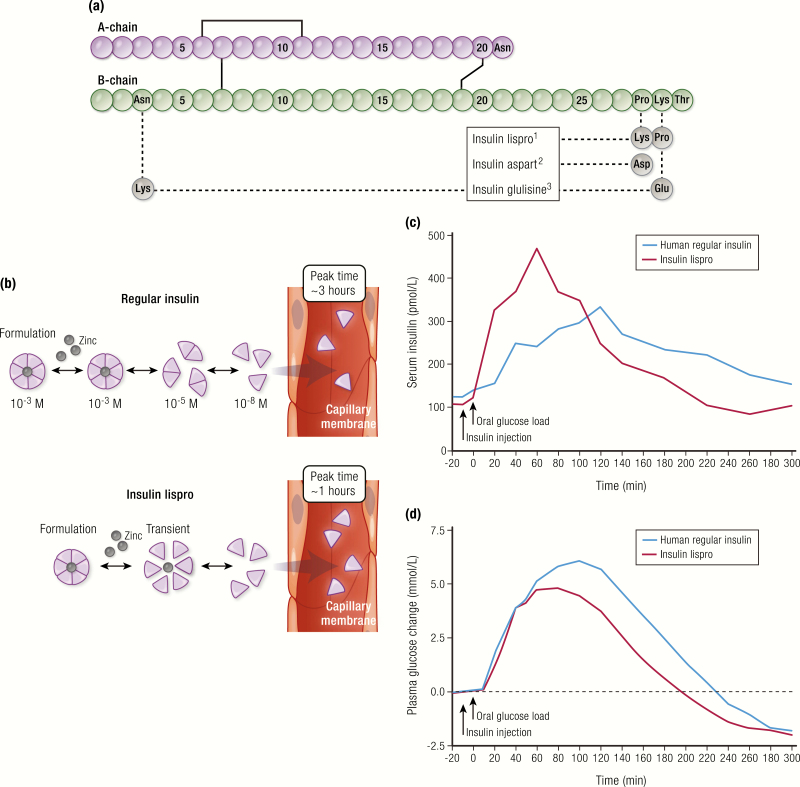
Rapid-acting insulins. **A:** Amino acid changes in the structures of rapid-acting insulin analogs. **B:** Quicker hexamer dissolution in the interstitial space of insulin lispro compared to regular insulin. **C:** Pharmacokinetics profile of insulin lispro compared to human regular insulin, showing a faster onset of action and peak of action, and a shorter duration of action when 0.075 U/kg of insulin was injected 10 minutes before a 50 g ^13^C-glucose load after an overnight fast (N = 8 subjects with type 2 diabetes). **D:** Pharmacodynamics profile from the same study, showing ^13^C-labeled serum glucose concentration during the study, corrected for ^3^H-labeled baseline glucose. Data for (**C**) and (**D**) from Bruttomesso D, Pianta A, Mari A, Valerio A, Marescotti MC, Avogaro A, et al. Restoration of early rise in plasma insulin levels improves the glucose tolerance of type 2 diabetic patients. *Diabetes.* 1999;48(1):99–105.

When the insulin is injected into the SC space, it does not immediately enter the blood stream. The time to enter blood circulation is based on both molecular properties of the insulin used (described in the sections that follow) and the type of formulation employed (ie, hexameric solution, acidic formulation, suspension formulation, etc.). Only insulin monomers (and perhaps dimers) can pass through the capillary walls. If the insulin solution contains the hexameric form of insulin, the time to onset is dependent on the strength of the interactions that bind the insulin hexamers together in the SC environment. When these interactions are strong, it takes longer for the hexamers to break down into monomers to enter circulation, increasing both the time to onset and the duration of action. When the interactions are weak, generation of monomeric insulin is accelerated, reducing both the time to onset and the duration of action (Figs. 3, 5, 8, 9, 10). Thus, the insulin formulation (monomers or hexamers) and the modifications employed to control hexamer stability provide a means of altering exogenous insulin therapy for SC delivery (eg, rapid-acting, intermediate-acting, and long-acting).

## Physiological Insulin Secretion

Normal physiologic insulin secretion includes both a continuous basal (low-level) insulin secretion and an incremental postprandial secretion associated with meals. For endogenous insulin, there is a feedback mechanism controlling insulin secretion based on changes in the blood glucose level sensed by the pancreatic islets and other tissues (20). When insulin is injected exogenously, there is no such feedback loop to make the adjustment to the doses. In order to adjust exogenous insulin, one needs to measure blood glucose and carbohydrate intake, which would then guide insulin doses.

Early insulin replacement therapy required multiple daily injections of 1 type of insulin only, with the risk of over or undershooting the immediate requirement. This situation was complicated by the lack of home blood glucose monitoring technology, an advance that did not come until the late 1970s (21, 22). There was a recognized need for longer-acting insulin to provide better between-meal coverage, as well as a faster-acting/faster-dissipating insulin for mealtime use (the concept of basal/bolus therapy) (23).

The limitations of human regular insulin injection as a bolus insulin are illustrated in Fig. 3C, where the PK profiles of endogenously secreted insulin (in healthy volunteers) and SC-administered human regular insulin (in persons with type 1 diabetes) following a meal are shown (24). Relative to the endogenous insulin response (blue line), the time to an increase in serum insulin concentration for SC-administered human regular insulin (red line) is notably slower, and the duration of the insulin in serum is significantly protracted. Consequently, an injection of human regular insulin requires administration at least 30 to 60 minutes prior to the meal to match maximal activity and minimize prolonged exposure. This is not only inconvenient for most people but can also increase the risk of hypoglycemia both before and after a meal if the insulin injection is not timed appropriately for the meal. These limitations of human regular insulin as bolus insulin were overcome using biotechnology to design and engineer rapid-acting insulin analogs.

## Rapid-acting Analog Insulins

Insulin analogs are insulin molecules wherein the amino acid structure is altered through genetic engineering and recombinant DNA technology to change the PK and PD properties (time to onset, peak, and duration of action) compared to human regular insulin, while preserving the biological properties and stability of the insulin molecule.

The first insulin analogs were an attempt to improve the therapeutic experience with mealtime insulin therapy. Specifically, analogs and formulations were designed to accelerate the time-dependent hexamer dissociation process and increase the rate of absorption from the injection site, relative to human regular insulin. This effort resulted in the development of insulin lispro, approved by the US Food and Drug Administration (FDA) as Humalog^®^ (Eli Lilly and Company) in 1996 (25). Insulin lispro was followed by other rapid-acting insulin analogs, insulin aspart, the drug substance in NovoLog^®^ or NovoRapid^®^ (Novo Nordisk A/S) in 2000 (26), and insulin glulisine, the drug substance in Apidra^®^ (Sanofi S/A, Paris, France) in 2004 (27).

All rapid-acting insulin analogs vary from human insulin by 1 or 2 amino acids in the primary structure (Fig. 5A). In insulin lispro and insulin aspart, these variations reduce the strength of the interactions that hold insulin dimers together, allowing for faster dissociation of hexamers in the SC space and hence absorption of monomers (Fig. 5B). With insulin glulisine, the same destabilizing effect on hexamers has been postulated, but the molecule can be formulated in a monomeric/dimeric state and thereby eliminates the delay associated with hexamer dissociation (28). Collectively, these analogs and formulations result in a quicker onset of action, a quicker time to peak activity, and a shorter duration of action compared to human regular insulin, making them more optimal for mealtime administration (Fig. 5C, Table 1) (29).

**Table 1.** Time-action of rapid-acting insulin analogs versus human regular insulin.

Insulin Type	Brand name	Onset of Action (min)	Peak Action (hr)	Duration of Action (hr)
**Short-acting**				
Human regular insulin	Humulin^®^ R Novolin^®^ R Insuman^®^ R	30–60	2–4	5–8
**Rapid-acting**				
Insulin lispro	Humalog^®^, Admelog	15–30	0.5–2.5	≤5
Insulin aspart	Novolog^®^ (26)	15	1–3	3–5
Insulin glulisine	Apidra^®^	12–30	1.5	~5.3
**Faster rapid-acting**				
Faster insulin aspart	Fiasp^®^ (30)	~16–20	~1.5–2.2	~5–7
Inhaled human insulin	Afrezza^®^ (34)	~12	0.5–0.9	1.5–3

Time-action parameters taken from Beals et al (28) and IBM Micromedex^®^ Pharmaceutical Knowledge database, unless otherwise indicated.

**Figure 6.**
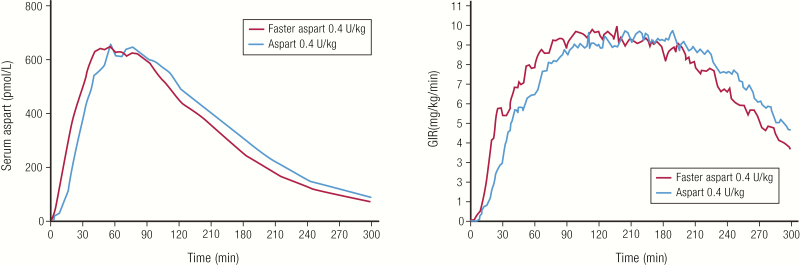
Faster-acting insulin aspart. **A:** Pharmacokinetics and PD profiles of faster aspart compared to aspart in 43 subjects with type 1 diabetes. Euglycemic clamp studies were conducted in a crossover fashion such that each subject was tested with 3 different doses of each insulin. The results for the 0.4 U/kg dose are shown. Data from Heise T, Stender-Petersen K, Hovelmann U, Jacobsen JB, Nosek L, Zijlstra E, et al. Pharmacokinetic and pharmacodynamic properties of faster-acting insulin aspart versus insulin aspart across a clinically relevant dose range in subjects with type 1 diabetes mellitus. *Clin Pharmacokinet.* 2017;56(6):649–660.

Newer, faster-acting insulins, called ultrarapid acting, are now entering the market. These insulins have an onset of action faster than rapid-acting analog insulins, allowing dosing to occur at the start of or even during a meal to better control postprandial glucose peaks. The first ultrarapid insulin, marketed as Fiasp^®^, was approved by the FDA in 2017 (30). Fiasp^®^ contains insulin aspart formulated with 2 additional excipients, L-arginine and niacinamide. L-arginine acts as a stabilizing agent, while niacinamide accelerates absorption at the site of injection (31). A comparison of the PK and PD profiles of Fiasp^®^ and insulin aspart showed an approximately 5- to 6-minutes faster onset of action with Fiasp^®^ (Fig. 6) (32). Fiasp^®^ is recommended to be injected at the start of a meal or within 20 minutes after starting a meal (30).

**Figure 7.**
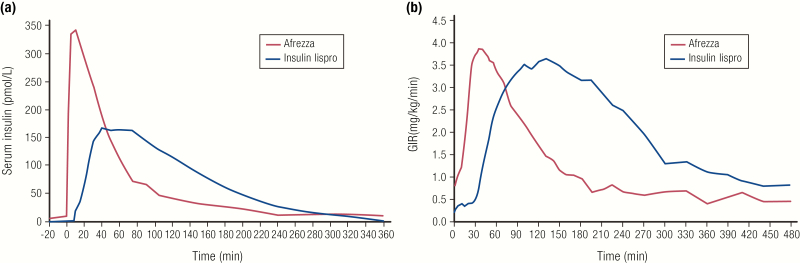
Afrezza^®^ inhaled insulin. **A:** Pharmacokinetics profile (baseline corrected serum insulin concentration) of inhaled Afrezza^®^ (8 units) compared to injected insulin lispro (8 units) in 12 subjects with type 1 diabetes. **B:** Pharmacodynamics profile of inhaled Afrezza^®^ (8 units) compared to injected insulin lispro (10 units) in 25 subjects with type 1 diabetes. Both studies were euglycemic clamp studies. Data from Heinemann L, Baughman R, Boss A, Hompesch M. Pharmacokinetic and pharmacodynamic properties of a novel inhaled insulin. *J Diabetes Sci Technol.* 2017;11(1):148–156.

Delivering insulin through inhalation instead of SC injection also allows for a faster onset of action. Afrezza^®^, approved in 2014, is currently the only inhaled insulin on the market in the United States. Unlike other rapid-acting and ultrarapid insulins, which are analogs of human insulin, Afrezza^®^ is a recombinant human regular insulin that has been formulated as a dry powder to be inhaled into the lungs via its dedicated device (33, 34). Afrezza^®^ has a rapid onset of action and a quick time to peak action, but a shorter duration of action compared with both human regular insulin or rapid-acting analog insulins, demonstrating how a change in the route of administration of human regular insulin can impact its PK and PD (Fig. 7, Table 1) (33).

In summary, the quicker time to onset and the short duration of rapid-acting and ultrarapid insulins mean that they can more closely mimic physiologic insulin secretion following a meal. Dosing can more precisely match a meal, lessen the postprandial spike in blood glucose, and reduce protracted exposure, thus lowering the risk of late hypoglycemia. However, the shorter the duration of bolus insulin, the more important it is to get the dose of basal insulin correct.

## Basal Insulin

Basal insulin refers to the constant, low level of insulin that is secreted to maintain stable blood glucose levels between meals and overnight. Basal insulin is an important component of insulin replacement therapy and is essential to appropriately suppress hepatic glucose production. Basal insulin, available in intermediate-acting and long-acting formulations, is often the first insulin used in treating people with type 2 diabetes.

### Intermediate-acting insulin

The first breakthrough in extending the duration of action of insulin came in 1936, when Hans Christian Hagedorn and B. Norman Jensen found that the addition of protamine (a protein obtained from the semen of trout) could prolong the effects of injected insulin (35). Protamine is a positive-charged protein that crystalizes with insulin hexamers, causing precipitation; this results in a suspension formulation of the insulin. When injected, the protamine/insulin crystals dissolve slowly, delaying the dissociation of insulin hexamers and thus slowing the absorption of insulin monomers into the circulation.

Precipitation of insulin with protamine or other agents was exploited in the 1930s and 1940s to make longer-acting insulin formulations. These formulations included globin insulin (with globin protein and zinc), surfen insulin (with surfen, which is bis-2-methyl-4-amino-quinolyl-6-carbamide), protamine zinc insulin (with protamine and zinc), and lente insulin (with zinc, in acetate buffer at the pH of blood) (36). Lente insulins include semilente, lente, and ultralente, with the extent of amorphous to crystalline insulin determining the duration of activity (37, 38). Protamine zinc insulin and lente insulins were widely used and are available today, mainly for veterinary practice.

**Figure 8.**
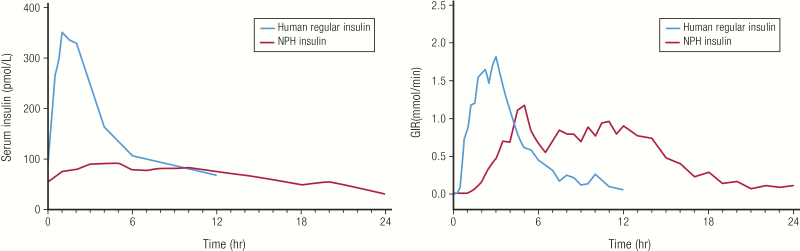
Neutral protamine hagedorn insulin. **A:** Pharmacokinetics and PD profiles of human NPH insulin (n = 6; 25 units SC) compared to human regular insulin (n = 10; 10 units SC) in healthy men. Subjects were fasted overnight prior to injection and kept under a euglycemic clamp for 24 hours or 12 hours, respectively. Data from Woodworth JR, Howey DC, Bowsher RR. Establishment of time-action profiles for regular and NPH insulin using pharmacodynamic modeling. *Diabetes Care.* 1994;17(1):64–69.

In 1950, neutral protamine hagedorn (NPH) insulin, also called isophane insulin, became available as the first intermediate-acting insulin, with a slower onset and longer duration of action compared with regular insulin formulations available at that time. This was helpful because it allowed patients to sleep through the night. Neutral protamine hagedorn insulin was originally animal-sourced; today NPH insulin is made with human regular insulin. In Fig. 8, we show how the PK and PD of NPH insulin and human regular insulin compare (39). The duration of action of 13 to 24 hours (Table 2) is longer than that of human regular insulin but not sufficient to mimic daily physiological basal insulin release in individuals with severe insulin deficiency; thus, NPH insulin needs to be administered twice-daily to achieve adequate basal coverage.

**Table 2.** Time-action of intermediate-acting and long-acting insulins (100 U/mL).

Insulin Type	Brand name	Onset of Action (hr)^*a*^	Peak Action (hr)	Duration of Action (hr)
**Intermediate-acting U-100**				
NPH insulin	Humulin^®^ N (40), Novolin^®^ N (41)	1–2	2–8	14–24
**Long-acting U-100**				
Insulin glargine	Lantus^®^ (43), Basaglar^®^	NA	No pronounced peak	24
Insulin detemir	Levemir^®^ (52)	NA	No pronounced peak	7.6–>24
Insulin degludec	Tresiba^®^ (59)	NA	No pronounced peak	42

^*a*^ The onset of action is not relevant for long-acting insulins.

As with human regular insulin, the time-action profile of NPH insulin can differ between individuals based on physiological factors. In addition, because NPH insulin is a precipitate with protamine and zinc, it needs to be resuspended by rolling it gently 12 to 15 times prior to injection (40, 41). If this resuspension process is not followed, it can add significantly to the day-to-day variability, which is substantial even in a controlled study (42).

### Long-acting insulin analogs

**Figure 9.**
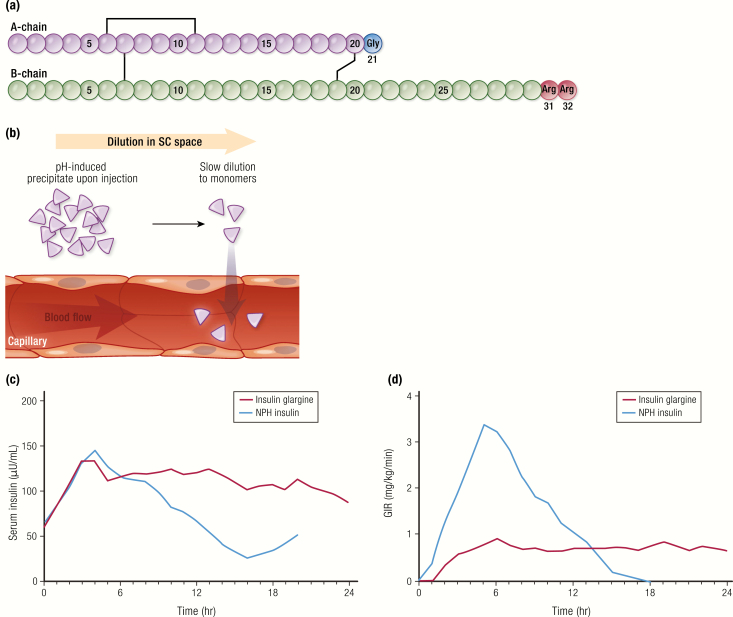
Insulin glargine. **A:** Amino acid structure of insulin glargine. **B:** Mechanism of protraction of insulin glargine: pH-induced precipitation at the SC space. **C and D:** Pharmacokinetics and PD profiles of insulin glargine U-100 compared to NPH insulin (each 0.3 U/kg) from a euglycemic clamp study in 20 subjects with type 1 diabetes. Data from Lepore M, Pampanelli S, Fanelli C, Porcellati F, Bartocci L, Di Vincenzo A, et al. Pharmacokinetics and pharmacodynamics of subcutaneous injection of long-acting human insulin analog glargine, NPH insulin, and ultralente human insulin and continuous subcutaneous infusion of insulin lispro. *Diabetes*. 2000;49(12):2142–2148.

Given the limitations of NPH insulin in meeting basal insulin needs, biotechnology was again employed to develop the first long-acting insulin analog. Insulin glargine, marketed as Lantus^®^ (Sanofi S/A), was first approved in the United States in the year 2000 (43). Insulin glargine differs from human insulin in that the amino acid asparagine in position A21 was substituted with glycine, and 2 arginine (glargine) residues were added at positions B31 and B32 (Fig. 9A). The arginine additions shifted the isoelectric point to near neutral pH and the replacement of asparagine at A21 with glycine imparted enhanced chemical stability in a low pH solution (formulated at pH 4). Upon injection into the neutral pH of the SC space, insulin glargine undergoes pH-induced precipitation (Fig. 9B). The pH-induced precipitate dissolves slowly, providing a time-action profile with a flattened peak and a median duration of action of up to 24 hours (Table 2). Glargine is given once daily in most patients, but in some instances it may need to be given twice daily (44). The relatively flat PK profile of glargine compared to NPH insulin (Fig. 9C) demonstrates how protein engineering of insulin can provide a stable insulin formulation with a long-lasting, low-level PD profile similar to endogenous basal insulin (45).

**Figure 10.**
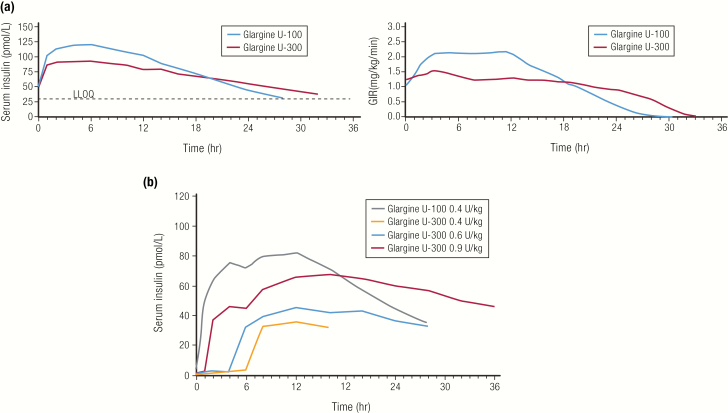
Nonbioequivalence of U-100 and U-300 glargine. **A:** Comparison of PK and PD profiles for insulin glargine U-100 and insulin glargine U-300 (0.4 U/kg each) in a euglycemic clamp study at steady state in 18 patients with type 1 diabetes. Data from Becker RH, Dahmen R, Bergmann K, Lehmann A, Jax T, Heise T. New insulin glargine 300 Units/mL provides a more even activity profile and prolonged glycemic control at steady state compared with insulin glargine 100 Units/mL. *Diabetes Care*. 2015; 38(4):637–643. **B:** Dose dependence of PK profile of insulin glargine U-300 in a euglycemic clamp study in 24 patients with type 1 diabetes. Data from Center for Drug Evaluation and Research. Clinical Pharmacology Review: Toujeo^®^ insulin glargine. 2014; https://www.accessdata.fda.gov/drugsatfda_docs/nda/2015/206538Orig1s000ClinPharmR.pdf. Accessed May 21, 2019.

A 3-fold concentrated version of insulin glargine was developed to prolong the duration of action when the results of 2 preliminary studies showed less diurnal variation in glucose-lowering activity with U-300 compared to the same dose of U-100 glargine (46). Insulin glargine U-300 (300 U/mL) was approved by the FDA in 2015 and marketed as Toujeo^®^ (Sanofi S/A) (47). The higher concentration of glargine delivered in the same volume further slows the dissolution of the glargine precipitate in the SC space, leading to a better basal insulin (48). The PK and PD profiles of the U-100 and U-300 formulations of insulin glargine are shown in Fig. 10A. The concentrated U-300 insulin glargine profile has a lower peak and a longer duration of action compared to U-100 insulin glargine at the same U/kg dose (46). The glucose-lowering effects of U-300 insulin glargine at steady state appear to be lower than U-100 insulin glargine at the same dose level (Fig. 10A) (46). In a study in type 1 diabetes, subjects needed 17.5% more U-300 than U-100 insulin glargine for the same glycemic control (49). In studies in type 2 diabetes, subjects needed an average of 12% more U-300 than U-100 insulin glargine for the same glycemic effect (50). Conversely, it is recommended that up to a 20% dose reduction may be needed to reduce the risk of hypoglycemia when switching from U-300 to U-100 insulin glargine (43). In addition, dose-dependent kinetics were observed with U-300 insulin glargine in people with type 1 diabetes, which is consistent with the mechanism described (Fig. 10B) (51).

**Figure 11.**
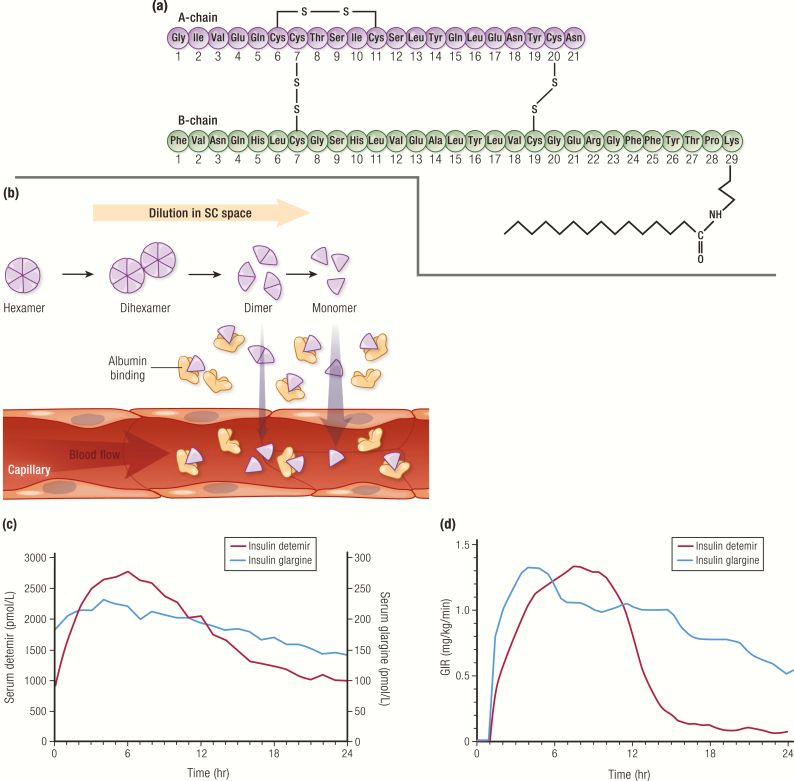
Insulin detemir. **A:** Amino acid structure of insulin detemir. **B:** Mechanism of protraction: di-hexamer formation in the SC space and binding to albumin. **C and D:** Pharmacokinetics and PD profiles of insulin glargine (n = 12, blue line) and insulin detemir (n = 12, red line) at steady state, from euglycemic clamp study following SC injection of 0.35 U/kg in patients with type 1 diabetes. Data from Porcellati F, Rossetti P, Busciantella NR, Marzotti S, Lucidi P, Luzio S, et al. Comparison of pharmacokinetics and dynamics of the long-acting insulin analogs glargine and detemir at steady state in type 1 diabetes: a double-blind, randomized, crossover study. *Diabetes Care*. 2007; 30(10):2447–2452.

Insulin detemir, marketed as Levemir^®^ (Novo Nordisk A/S), was the next long-acting basal insulin developed, approved in by the FDA in 2005 (52). The insulin detemir molecule has the amino acid threonine at B30 omitted and a 14-carbon fatty acid covalently attached to the lysine at B29 (Fig. 11A). Insulin detemir is formulated as a solution of hexamers at neutral pH. After injection and dilution of the preservative in the SC space, the fatty acyl side chain stabilizes the hexamers and promotes self-association of hexamers to dihexamers. This prolongs the persistence of insulin detemir at the injection site by slowing hexameric dissociation and subsequent monomeric absorption (Fig. 11B). The fatty acyl side chain also enables binding to serum albumin, a long-lived plasma protein, thus slowing the disposition of detemir to peripheral tissues and clearance from the body (53).

Compared to NPH insulin, insulin detemir has a slower onset of action, with a peak at 6 hours and a duration of action up to 24 hours (Table 2). Compared to insulin glargine however, insulin detemir has a shorter duration of action, and therefore may need to be given twice a day, particularly in patients with type 1 diabetes (Fig. 11C, Table 2) (54). Those with type 2 diabetes, especially with a higher body mass index, may also need higher doses of insulin detemir compared to insulin glargine or NPH insulin (55, 56). Injection-to-injection glycemic variablity is greatly reduced with insulin detemir compared to insulin glargine (which precipitates on injection) or NPH insulin (which is a suspension of precipitated insulin), given that detemir remains in solution form (56–58).

**Figure 12.**
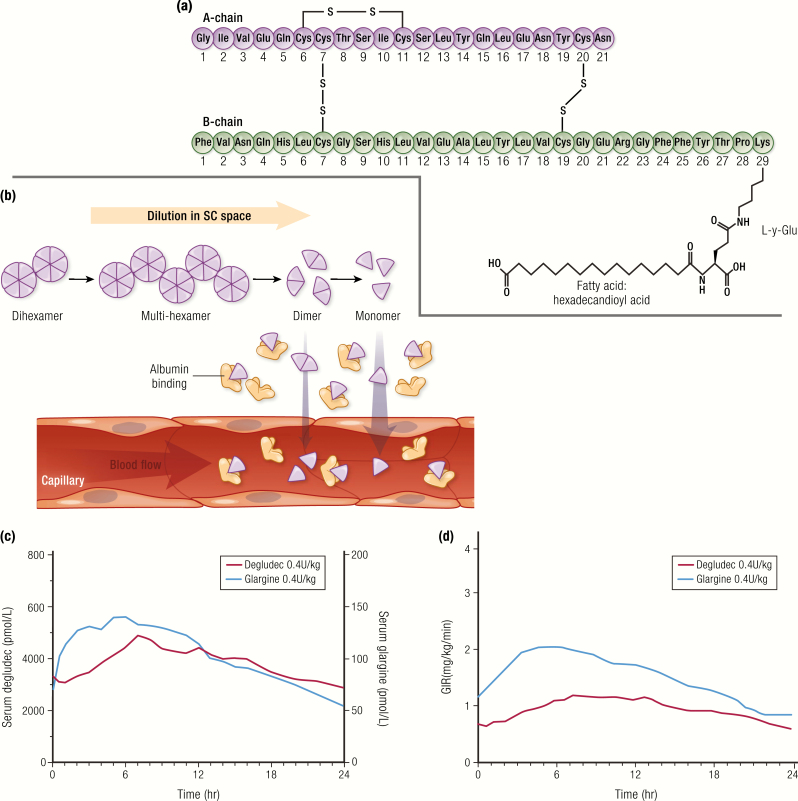
Insulin degludec. **A:** Amino acid structure of insulin degludec. **B:** Mechanism of sustained release exploits multihexamer formation and binding to albumin. **C and D:** Comparison of PK and PD of insulin degludec (N = 22; 0.4 U/kg) and insulin glargine (N = 22; 0.44 U/kg) at steady state from a euglycemic clamp study in subjects with type 1 diabetes. Data from Heise T, Hovelmann U, Nosek L, Hermanski L, Bottcher SG, Haahr H. Comparison of the pharmacokinetic and pharmacodynamic profiles of insulin degludec and insulin glargine. *Expert Opin Drug Metab Toxicol*. 2015;11(8):1193–1201.

The continued search for a basal insulin with a longer than 24-hour duration of action and a flatter insulin profile led to the development of insulin degludec, marketed as Tresiba^®^ (Novo Nordisk A/S), approved by the FDA in 2015 (59). Insulin degludec is the longest-acting insulin analog on the market today. In the insulin degludec molecule, the B30 threonine is omitted and a side chain is attached to the B29 lysine consisting of glutamic acid and a 16-carbon fatty acid with a terminal carboxylic acid group (Fig. 12A). The diacyl side chain of insulin degludec, in the presence of zinc and phenolic preservative, promotes association into dihexamers under formulation conditions. After injection and dilution of the phenolic preservative in the SC space, the dihexamers self-associate, forming multihexamer complexes (60). This higher-order structure significantly slows hexameric dissociation and subsequent monomer absorption (Fig. 12B). The binding of monomers to albumin in the circulation also slows the disposition of degludec to peripheral tissues and clearance from the body, leading to a protracted time-action profile (61). Insulin degludec has a duration of action of at least 42 hours at steady state (Table 2) (59).

The PK and PD profiles of insulin degludec compared to U-100 insulin glargine in subjects with type 1 diabetes are shown in Fig. 12C (62). The time-action profile of insulin degludec at steady state with once-daily dosing is characterized by a near-constant plasma level with minimal peak to trough, reducing day-to-day variability (61). Outcomes from flexible dosing studies suggest that the daily injection time of insulin degludec can be varied without compromising glycemic control or safety (63). The long duration of action of degludec, however, needs to be kept in mind when a patient is made nil per os for a surgical procedure or when switching to a different basal insulin, since there could be residual insulin action for up to 42 hours (64). This transition is particularly complex when switching from degludec to insulin pump therapy (64).

It should be noted that the PK figures for both insulin detemir and insulin degludec (Figs. 11C and 12C) show serum concentrations that are much higher than those required for insulin glargine to achieve the same glycemic effect (both PK figures have separate vertical axes, 1 for each insulin). This is due to the binding affinity of insulin detemir and insulin degludec for albumin. The fact that most of the circulating insulin detemir or insulin degludec is bound to albumin reduces the amount of free drug available for engagement with the insulin receptor, whereas insulin glargine is all available as free drug to bind the insulin receptors (65). In practice, these differences are mostly accounted for, since all insulins are standardized to the same international units.

Overall, the predictable time-action profiles of long-acting insulin analogs, with flattened peaks and prolonged duration of action, do a better job of mimicking endogenous basal insulin secretion compared to intermediate-acting insulins, such as NPH. These properties of the long-acting analogs may also reduce the risk of hypoglycemia, especially nocturnally (48, 66, 67).

## Insulin Mixtures

Patients sometimes mix a short-acting or rapid-acting mealtime insulin and intermediate-acting NPH insulin to limit the number of daily injections, and yet provide both basal and bolus coverage, a process referred to as “free mixing.” Although free mixing is still practiced, premixed insulin formulations have gained in popularity in some parts of the world. These premixed insulin formulations add to the convenience for some patients and also prevent mixing errors.

**Figure 13.**
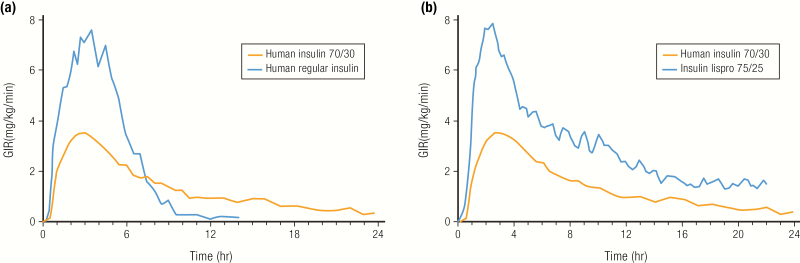
Premix insulins. **A:** Pharmacodynamics profiles for a 0.3 U/kg dose of an insulin premix (NPH insulin 70%, human insulin 30%) and a 0.3 U/kg dose of human regular insulin from a euglycemic clamp study in 18 healthy subjects. Data from Eli Lilly and Company. Humulin^®^ 70/30 Prescribing Information. 2018; http://pi.lilly.com/us/HUMULIN-7030-USPI.pdf. Accessed April 4, 2019. **B:** Pharmacodynamics profiles of human insulin premix (NPH insulin 70%, human insulin 30%; 0.3 U/kg) (N = 18) or an analog insulin premix (NPL insulin 75%, insulin lispro 25%; 0.3 U/kg) (N = 30) from a euglycemic clamp study in healthy subjects. Data from Heise T, Weyer C, Serwas A, Heinrichs S, Osinga J, Roach P, et al. Time-action profiles of novel premixed preparations of insulin lispro and NPL insulin. *Diabetes Care*. 1998;21(5):800–803.

Premixed insulins are predicated on being able to blend the basal insulin with the mealtime insulin in the same vial or pen, with both retaining their respective PK properties during storage. Premixed insulins can be based on human insulins or analog insulins. With human insulin mixtures, NPH insulin (70%) is combined with human regular insulin (30%). Some of the regular insulin binds to the NPH insulin in the formulation, but the binding reverses once the mixture is injected. Given the kinetics of human regular insulin in the mixture, the premix needs to be injected 30 to 45 minutes before the meal (68). The PD profiles of a human 70/30 mixture compared to human regular insulin in healthy subjects is shown in Fig. 13A (68). The mixture provides a similar onset of action compared to U-100 regular insulin, albeit with a lower peak (probably a result of the smaller dose of regular insulin in the mixture) but a longer duration of action from the NPH component.

Rapid-acting insulin analogs are also provided as premixes (Table 3). Mixtures of insulin lispro, with its protamine suspension, neutral protamine lispro (NPL) insulin (which was specifically developed for mixing with insulin lispro), are available as 75/25 and 50/50 percent mixtures of NPL/insulin lispro (Humalog^®^ Mix 75/25 (69) and Humalog^®^ Mix 50/50 (70)); a similar premix is available for insulin aspart, with its protamine suspension (Novolog^®^ 70/30 (71)). The PD profiles of a 75/25 mixture of insulin lispro with NPL insulin are compared to a human insulin 70/30 premix in healthy subjects (Fig. 13B) (72).

**Table 3.** Premixed insulins currently available in the United States.

Brand Name	Components	Peak Action (min)
**Human insulin mixtures**		
Humulin^®^ 70/30 Novolin^®^ 70/30	70% human NPH insulin; 30% human regular insulin	132 (68)
**Analog insulin mixtures**		
Humalog^®^ mix 75/25	75% insulin lispro protamine suspension; 25% insulin lispro	94 (69)
Humalog^®^ mix 50/50	50% insulin lispro protamine suspension; 50% insulin lispro	81 (70)
NovoLog^®^ mix 70/30	70% insulin aspart protamine suspension; 30% insulin aspart	162 (71)
Ryzodeg^®^ 70/30	70% insulin degludec; 30% insulin aspart	72 (73)

Please consult package inserts for further information.

**Figure 14.**
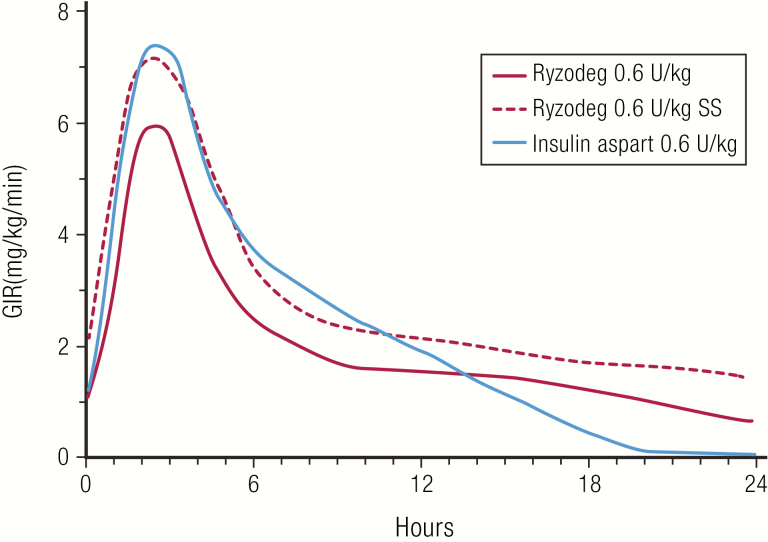
Ryzodeg^®^ (insulin aspart 30%, insulin degludec 70%). Pharmacodynamics profiles of single doses of Ryzodeg^®^ and Novolog^®^ (insulin aspart) at 0.6 U/kg from a euglycemic clamp study in 31 subjects with type 1 diabetes. Also shown for comparison is the PD profile of Ryzodeg^®^ 0.6 U/kg from a euglycemic clamp study in 22 subjects with type 1 diabetes taking insulin degludec for 5 days prior to the study to achieve steady state (SS). Data from Heise T, Nosek L, Klein O, Coester H, Svendsen AL, Haahr H. Insulin degludec/insulin aspart produces a dose-proportional glucose-lowering effect in subjects with type 1 diabetes mellitus. *Diab Obes Metab*. 2015;17(7):659–664.

A mixture of rapid-acting insulin aspart (30%) with long-acting insulin degludec (70%), marketed as Ryzodeg^®^ (Novo Nordisk A/S), was introduced in 2015 (73). Ryzodeg^®^ is the only analog–analog insulin mixture and is formulated without protamine (Table 3). In subjects with type 1 diabetes, Ryzodeg^®^ had a longer duration of action compared with Novolog^®^ 70/30, especially at steady state (74–76). The PD profiles of Ryzodeg^®^ single dose compared to insulin aspart single dose, are shown in Fig. 14, as well as Ryzodeg^®^ single dose given to subjects who had achieved steady state on insulin degludec (75, 76).The advantage of analog-based mixtures is that they can be timed in closer proximity to the meal, adding to the convenience for the patient.

Premix insulin formulations are a well-established treatment for type 2 diabetes and can be administered shortly before meals as often as once, twice, or thrice daily (77, 78). These products have the advantage of fewer injections compared to basal/bolus therapy. Clinically, these insulins are best suited for those individuals with consistency in food intake and timing of their meals, as the individual insulin components cannot be adjusted separately.

## Concentrated Insulins

Concentrated insulins have been developed for a variety of purposes (79). With the epidemic of obesity and type 2 diabetes, the number of people requiring high doses of insulin has been growing. Although concentrated insulins were not developed primarily for people requiring high dose insulin therapy, they do have the ability to deliver more insulin with lower volume, fewer injections, and less pain at the injection site compared to U-100 insulin, potentially improving adherence in this population (80–82). A summary of concentrated insulins available today is given in Table 4.

**Table 4.** Concentrated insulins currently available in the United States.

Insulin Name	Concentration	Brand Name	Insulin Type	Notes
**Bioequivalent to reference 100 U/mL**				• Equivalent time-action profiles. • No dose adjustment needed.
Insulin lispro	200 U/mL	Humalog^®^	Prandial analog	
Insulin degludec	200 U/mL	Tresiba^®^	Basal analog	
**Nonbioequivalent to reference 100 U/mL**				• Different time-action profiles. • Dose adjustment needed for U-300. • No dose adjustment required for U-500R.
Insulin glargine	300 U/mL	Toujeo^®^	Basal analog	
Human regular insulin	500 U/mL	Humulin^®^ R U-500	Prandial/basal human	

The first concentrated insulin developed was U-500 (5x concentrated animal-sourced insulin), which was needed to overcome insulin resistance associated with high concentrations of anti-insulin antibodies from animal-sourced insulins (83). With the advent of recombinant DNA technology, human regular U-500 insulin, marketed as Humulin^®^ R U-500 (Lilly) was introduced without the same immunogenic properties as its animal-sourced counterpart. U-500 insulin retains the bolus properties of regular insulin but also has a longer duration of action (84). U-300 insulin glargine (discussed above) was developed to prolong the duration of action of insulin glargine, while U-200 formulations of insulin lispro and insulin degludec were developed to allow for lower volumes of the drug to be delivered.

**Figure 15.**
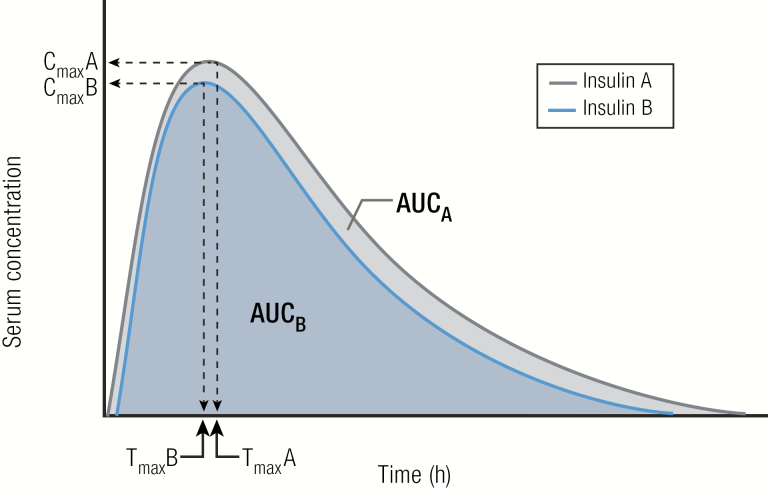
Test for bioequivalence of 2 insulins. Pharmacokinetics parameters of the test insulin (**B**), usually AUC and C_max_ (peak concentration reached), must lie within a prespecified range of the reference insulin (**A**) to be considered bioequivalent when administered at the same molar dose under the same conditions.

**Figure 16.**
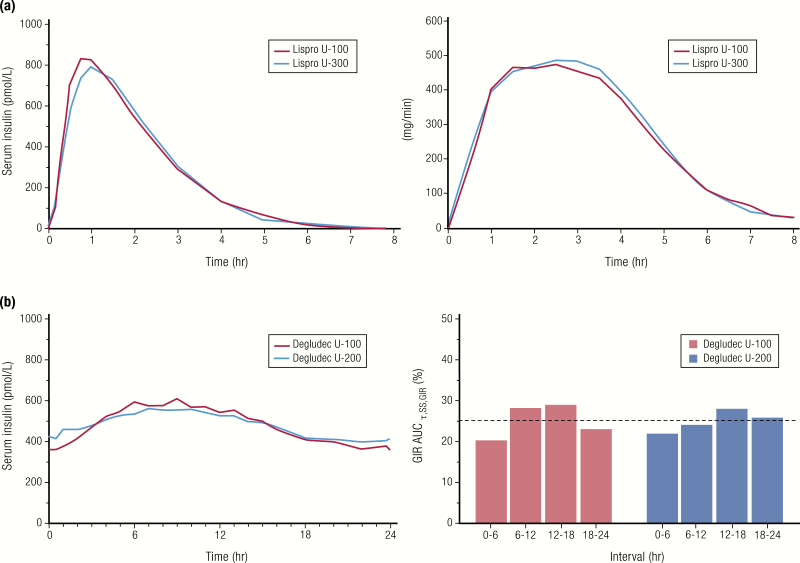
Bioequivalent insulins. **A:** Comparison of PK and PD profiles for insulin lispro U-100 and insulin lispro U-200 from a euglycemic clamp study in 38 healthy subjects (SC administration of 20 U). Data from de la Pena A, Seger M, Soon D, Scott AJ, Reddy SR, Dobbins MA, et al. Bioequivalence and comparative pharmacodynamics of insulin lispro 200 U/mL relative to insulin lispro (Humalog^®^) 100 U/mL*. Clin Pharmacol Drug Dev*. 2016;5(1):69–75. **B:** Comparison of PK and PD profiles for insulin degludec U-100 and insulin degludec U-200 from a euglycemic clamp study at steady state (N = 33 patients with type 1 diabetes; 0.4 U/kg given once daily with insulin aspart at mealtimes). Data from Korsatko S, Deller S, Koehler G, Mader JK, Neubauer K, Adrian CL, et al. A comparison of the steady-state pharmacokinetic and pharmacodynamic profiles of 100 and 200 U/mL formulations of ultralong-acting insulin degludec. *Clin Drug Investig*. 2013;33(7):515–521.

In practical terms, however, switching someone from a U-100 insulin to a more concentrated insulin requires knowledge of the bioequivalence of the concentrated insulin to its U-100 counterpart to determine the correct dose and timing. Bioequivalence is defined by the FDA as the “absence of significant difference in rate and extent to which active ingredient/moiety in pharmaceutical equivalents or alternatives becomes available at the site of drug action when administered at the same molar dose under similar conditions in an appropriately designed study” (85). Bioequivalence for insulins implies equivalent efficacy (PK and PD) when delivering the same units in a reduced volume. As shown in Fig. 15, the 90% confidence interval (CI) for the ratio of PK parameters (area under the curve [AUC], C_max_) for the 2 insulins (B/A) must lie within the prespecified acceptance interval, say 80% to 125%, to insure comparable in vivo performance. For example, U-200 insulin lispro has been determined to be bioequivalent to U-100 insulin lispro, meaning that the volume of insulin can be reduced by 50% for U-200 with similar results (Fig. 16A) (86). Similarly, insulin degludec U-200 has been determined to be bioequivalent to insulin degludec U-100 (Fig. 16B) (87). This allows for a 1:1 insulin dose conversion, on a unit basis, when switching from U-100 to a U-200 formulation of insulin lispro or insulin degludec.

**Figure 17.**
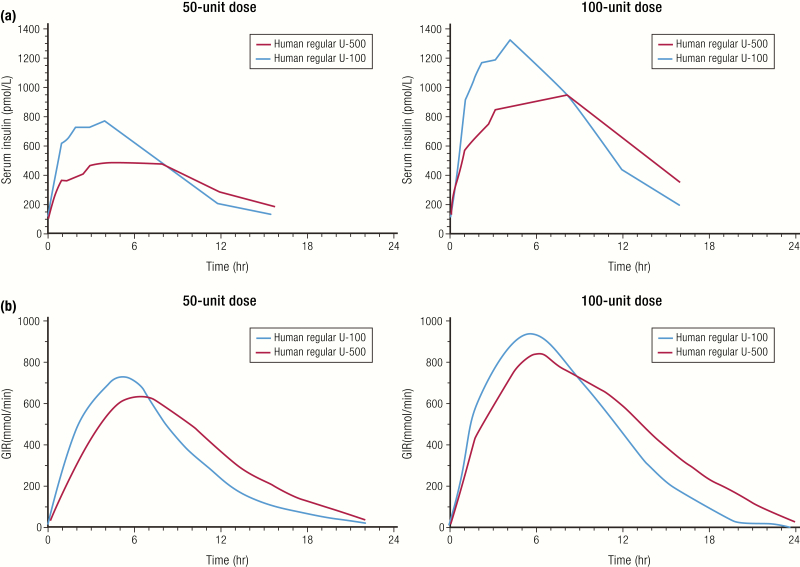
Nonbioequivalent insulins. Pharmacokinetics profiles (**A**) and PD profiles (**B**) of 2 doses each of human regular U-100 and U-500 insulins from a euglycemic clamp study in 24 healthy obese subjects. Data from de la Pena A, Riddle M, Morrow LA, Jiang HH, Linnebjerg H, Scott A, et al. Pharmacokinetics and pharmacodynamics of high-dose human regular U-500 insulin versus human regular U-100 insulin in healthy obese subjects. *Diabetes Care*. 2011;34(12):2496–2501.

The more concentrated insulins, insulin glargine U-300 (Toujeo^®^) and human insulin U-500R, are not bioequivalent to their U-100 counterparts. The comparative PK and PD profiles of U-100 and U-300 insulin glargine formulations are discussed above (Fig. 10A and 10B). U-500R insulin is the most concentrated insulin on the market today, with a 5-times higher concentration compared to U-100 human regular insulin. The PK and PD profiles for 2 doses each of U-100R and U-500R insulins are shown in Fig. 17 (84, 88). The time to peak action was similar for both formulations at the 50-unit and 100-unit doses; however, the peak action was lower and the overall duration of action was prolonged for U-500R. Both U-100R and U-500R formulations had a similar onset of action of less than 15 minutes with both doses. Given the similar onset and peak action to U-100R, the U-500R formulation provides prandial (mealtime) coverage while its extended duration of action provides basal insulin properties. This attribute facilitates the use of U-500R as insulin monotherapy, which was tested and shown to significantly improve HbA1c in patients with type 2 diabetes inadequately controlled on high doses of U-100R therapy (> 200 units/day) (89). While total insulin exposure (AUC_insulin_ and G_tot_) was not statistically different between U-500R and U-100R, supporting unit dose equivalency (equipotency) (84, 90, 91), they are not bioequivalent and have different time-action profiles (Fig. 17). When starting a person on U-500R, dose at initiation should be determined based on current and targeted glycemic goals to optimize safety and efficacy.

## Biosimilars/Follow-on Biologics

As the technology to develop insulin at a commercial scale has improved, not only have there been significant advances in the development of newer insulins with novel kinetic profiles, but there has also been an increase in the competitive landscape with the development and marketing of existing insulins by other manufacturers. Unlike small chemical compounds, such as aspirin, insulin is a biologic medicine, a term that describes a large molecule (usually a protein) derived from microorganisms or human or animal cells using biotechnology. Where for small chemical compounds a follow-on compound would be referred to as “generic,” the terminology generally used to describe a follow-on biologic molecule is “biosimilar,” although for insulin products the terminology is less clear. To facilitate the development of biological compounds similar to those already on the market, both the FDA and the European Medicines Agency (EMA) have issued guidelines.

Per the FDA, a “biosimilar” is a biological product that is highly similar to a US-licensed reference biological product notwithstanding minor differences in clinically inactive components, and for which there are no clinically meaningful differences between the biological product and the reference product in terms of the safety, purity, and potency of the product (92, 93). The EMA has a similar definition (94). However, to be designated as a biosimilar, the FDA requires that the FDA-licensed reference product must have been approved as a biologic. Since recombinant insulin was originally approved as a drug and not a biologic in the United States, there was no pathway to approve a “biosimilar” insulin. Rather, the term “follow-on biologic” was used for insulins that were developed to be similar to an existing approved insulin. The pathway for follow-on biologic approval requires data that are generated for the follow-on product as well as data that bridge to the safety and efficacy of the existing approved insulin. Follow-on insulin products generally were referred to as “biosimilars” in other parts of the world (94). As of March 2020, insulin products will be considered as approved under the biologics pathway in the United States, and therefore will become eligible for approval as biosimilars (93).

**Figure 18.**
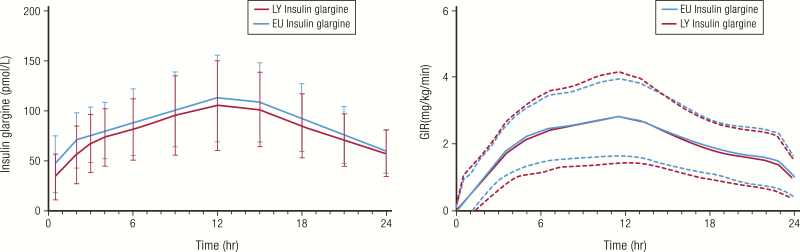
Example of similar PK and PD profiles of biosimilar insulin glargine products (Basaglar^®^ and Lantus^®^). Healthy subjects (n = 80) received 0.5 units/kg of insulin in a euglycemic clamp study. Data are mean and standard deviation. Abbreviations: EU IGlar, Lantus^®^; LY IGlar, Basaglar^®^. Data from Linnebjerg H, Lam EC, Seger ME, Coutant D, Chua L, Chong CL, et al. Comparison of the pharmacokinetics and pharmacodynamics of LY2963016 insulin glargine and EU- and US-approved versions of Lantus^®^ insulin glargine in healthy subjects: three randomized euglycemic clamp studies. *Diabetes Care*. 2015;38(12):2226–2233.

A comparison of the PK and PD properties of insulin glargine from 2 manufacturers is shown in Fig. 18 (95). In the study shown, the test product (LY IGlar, Basaglar^®^) met the criterion for similarity with the reference product (EU IGlar, Lantus^®^) based on the regulatory guidance that the 90% CI for the ratio of the geometric means of the of PK and PD parameters for the 2 products are contained within predefined acceptance limits of 80% to 125 % (96).

There are currently 4 follow-on biologic/biosimilar insulins that have been approved (Table 5), with others currently under development. Follow-on biologics/biosimilars increase the number of treatment options available to patients, prescribers, and payers. This may increase the accessibility of treatments, and potentially reduce costs.

**Table 5.** Currently available follow-on biologic or biosimilar U-100 insulins.

Product Name	Manufacturer	Reference Product	Molecule	Approvals
**Follow-on biologic/biosimilar insulins**				
Basaglar^®^, Abasaglar^®^	Eli Lilly	Lantus^®^	Insulin glargine	FDA 2015; EMU 2014
Basalin^®^	Gan & Lee	Lantus^®^	Insulin glargine	China 2005
Semglee^®^	Biocon/Mylan	Lantus^®^	Insulin glargine	FDA under review; EU, Australia 2018
Admelog^®^	Sanofi	Humalog^®^	Insulin lispro	FDA 2017

## Insulin Accessibility

Although a variety of insulins are available that allow therapy to be tailored to individual patients, many patients are experiencing issues related to accessing and affording their therapy (97, 98). Access not only to insulin but also to glucose monitoring supplies is a problem worldwide, with many contributing factors that vary geographically (97). With regard to the cost of insulin in the United States, a working group of the American Diabetes Association has extensively discussed this issue (98). Some of the key recommendations of this working group were: (1) the current payment system should rely less on rebates, discounts, and fees based on list price; (2) health plans should ensure that people with diabetes can access their insulin without undue administrative burden or excessive cost; and (3) pharmacy benefit managers and payers should use rebates to lower the costs for insulin at the point of sale for people with diabetes. In addition, the group recommended that prescribers should be trained in prescribing all forms of insulin preparations and consider prescribing the lowest-cost insulin that can safely and effectively achieve the patient’s individual glycemic goals. International organizations, government agencies, and industry are actively seeking solutions to alleviate these problems.

## Technology in Insulin Delivery

For many years after the discovery of insulin, a vial and syringe were the only delivery options available. Today, insulin pens, either disposable with prefilled insulin cartridges or reusable with replaceable cartridges, are widely used for injection insulin therapy. Insulin pens offer benefits to patients, including more accurate dosing, ease of use, more social acceptability, and less time to prepare and administer injections (99).

However, despite vast improvements in the chemistry of insulin molecules to facilitate physiological insulin replacement, exogenous insulin therapy still requires dose calculations and glucose monitoring. To facilitate this process, “connected” pens are being developed that link to smartphone-based diabetes management apps (100). Such connected devices are not only able to record the time and amount of insulin delivered, but the apps can be connected to blood glucose and continuous glucose monitoring (CGM) devices and help provide not only comprehensive diabetes management data but in some cases also insulin dose recommendations. To mimic physiological insulin delivery even better, the FDA has approved 2 “hybrid closed loop systems,” which allow for automated adjustment to the basal insulin infusion rates (Minimed 670G system) and also automated bolus corrections (t:slim X2 with Control IQ algorithms) based on CGM readings (101–103).

## Summary and Conclusions

Replacement insulin therapy should mimic the body’s own insulin response as closely as possible. Great strides have been made in achieving this goal through innovation and the use of biotechnology, including recombinant DNA technology, protein engineering, formulation strategies, and advances in manufacturing. Of course, true insulin replacement requires the feedback control on insulin levels, such as is provided naturally by healthy beta cells in response to changes in blood glucose. For this, we have evolved from using episodic self-monitored blood glucose values to CGM technology, which could enable more accurate, feedback-based insulin replacement with pens and hybrid closed-loop pump systems. Advances in insulin’s molecular properties through new analogs, coupled with advances in glucose monitoring and dosing algorithms, will continue to make insulin therapy safer and more effective for people with diabetes.

## Additional Information:

***Disclosure Summary:*** I.B.H. receives research grants from Medtronic Diabetes and Insulet Corporation, and serves as a consultant for Abbott Diabetes Care, Bigfoot, and Roche. E.E.W. serves as a consultant for Abbott Diabetes, Astra Zeneca, Bayer, Boehringer Ingelheim, Eli Lilly, Mannkind, Merck, Sanofi US, and Voluntis, and serves on the speaker’s bureau for Abbott Diabetes, Boehringer Ingelheim, and Eli Lilly. R.J., C.J.A., and J.M.B. are employees and shareholders of Eli Lilly and Company.

***Data Availability:*** Data sharing is not applicable to this article as no datasets were generated or analyzed during the current study.
